# Non-covalently Functionalized Graphene Oxide-Based Coating to Enhance Thermal Stability and Flame Retardancy of PVA Film

**DOI:** 10.1007/s40820-018-0190-8

**Published:** 2018-02-16

**Authors:** Wenhua Chen, Pengju Liu, Lizhen Min, Yiming Zhou, Yuan Liu, Qi Wang, Wenfeng Duan

**Affiliations:** 1 State Key Laboratory of Polymer Materials Engineering, Polymer Research Institute of Sichuan University, Chengdu, 610065 People’s Republic of China; 2 State Key Laboratory of Special Functional Waterproof Materials, Beijing Oriental Yuhong Waterproof Technology Co. Ltd., Beijing, 101300 People’s Republic of China

**Keywords:** Graphene, Non-covalent functionalization, Layer-by-layer, Flame retardant, Poly(vinyl alcohol) (PVA)

## Abstract

The synergistic effect of conventional flame-retardant elements and graphene has received extensive attention in the development of a new class of flame retardants. Compared to covalent modification, the non-covalent strategy is simpler and expeditious and entirely preserves the original quality of graphene. Thus, non-covalently functionalized graphene oxide (FGO) with a phosphorus–nitrogen compound was successfully prepared via a one-pot process in this study. Polyethyleneimine and FGO were alternatively deposited on the surface of a poly(vinyl alcohol) (PVA) film via layer-by-layer assembly driven by electrostatic interaction, imparting excellent flame retardancy to the coated PVA film. The multilayer FGO-based coating formed a protective shield encapsulating the PVA matrix, effectively blocking the transfer of heat and mass during combustion. The coated PVA has a higher initial decomposition temperature of about 260 °C and a nearly 60% reduction in total heat release than neat PVA does. Our results may have a promising prospect for flame-retardant polymers.

**Electronic supplementary material:**

The online version of this article (10.1007/s40820-018-0190-8) contains supplementary material, which is available to authorized users.

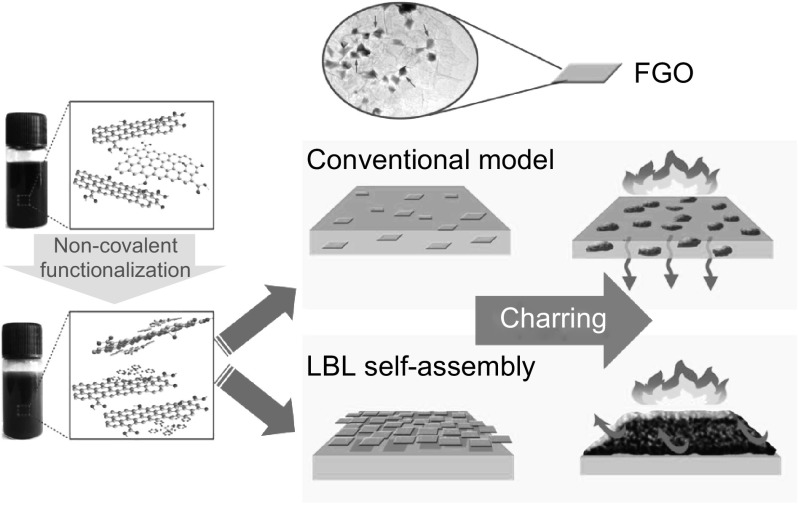

## Highlights

- Functionalized graphene oxide (FGO) containing phosphorus–nitrogen compound was prepared via a non-covalent strategy.
- The multilayer FGO-based coating was deposited on a poly(vinyl alcohol) (PVA) film using the layer-by-layer assembly technique.
- A significant synergistic effect between the FTO and conventional flame-retardant elements enhances thermal stability and fire retardancy of the coated PVA film.

## Introduction

Multifunctional polymer films have received much attention owing to their wide potential use in both research and practice applications. Conducting polymers such as polyaniline (PANI) [1, 2] or polypyrrole (PPy) [3–5] thin films with remarkable storage capacity have been widely applied as supercapacitor electrodes. Polyamide (PA) thin-film composite is considered a promising candidate for filtration membrane for water purification. A series of film products are also used in solar cells [6], sensors [7], and heavy metal detoxification [8]. In addition, satisfactory flame-retardant property of polymeric membranes is crucial for their applications in numerous fields. In particular, as low-dimensional materials, the thin wall structure of polymer membranes leads to much higher combustibility compared to three-dimensional polymer products. Incorporation of flame-retardant additives into a film matrix is a conventional approach to reducing its flammability [9, 10]. However, the addition of flame retardants produces interior defects that deteriorate the film-forming ability and mechanical properties. Therefore, fabrication of high-performance flame-retardant polymer films via the conventional strategy remains a challenge worldwide.

Graphene, a two-dimensional carbon material, has remarkable properties such as superior mechanical strength and high thermal and electrical conductivities [11–14]. These intrinsic properties of graphene have gained enormous interest in various fields including solar cells [15], flexible electrodes [16, 17], ultrasensitive sensors [18, 19], and reinforced nanocomposites [20, 21]. Besides, in the last few decades, graphene has been extensively applied in various polymer systems for improving their flame-retardant properties [22–24]. Due to its layered structure, graphene acts as a barrier retarding heat release and blocks the diffusion of pyrolysis products and the transfer of oxygen. However, because of combustibility in air atmosphere [25–27], satisfactory flame retardancy is hard to achieve via incorporation of individual graphene into the polymer matrix.

Flame-retardant materials contain one or more flame-retardant elements such as phosphorus [28, 29], nitrogen [30, 31], and silicon [32]. These flame-retardant elements may be added in the form of an additive or chemically incorporated into the structure of the materials. Therefore, fabricating hybrid graphene-based compounds containing conventional flame-retardant elements may be an effective way to improving the fire resistance efficiency of graphene. In our previous work, owing to the barrier effect of graphene sheets in the initial combustion stage, a slower chemical charring behavior of phosphorus flame retardant (PFR) was observed. Thus, the system maintained a good shielding action during the entire process because of the double barrier effect of PFR-graphene oxide (GO) [33]. Chiang et al. [34] prepared a novel phosphorus-containing reduced GO (rGO) flame retardant (DOPO-rGO) via a direct reaction between them. Resulting from the synergistic effect of DOPO-rGO, the epoxy resin was endowed with excellent flame retardancy.

Hexachlorocyclotriphosphazene (HCCP), a phosphorus–nitrogen compound, was chemically grafted onto the surface of GO. HCCP catalyzed the char formation from polymers, and graphene was protected from oxygen after being encapsulated by the HCCP-induced char. Thus, graphene did not burn out and acted as a graphitic char in the condensed phase [23, 25]. In this case, functionalized GO (FGO) with phosphorus–nitrogen elements will offer significantly enhanced flame retardancy [35–37]. It is worth noting that the oxygen-containing functional groups on the basal plane and along the edges of GO, the reactive sites in the chemical modification, will always be partially replaced. The hydrophilicity of FGO deteriorates to a certain extent, which renders its exfoliation in water into individual GO sheets forming a stable colloidal suspension difficult [33]. Since homogeneous suspension of graphene sheets in water is crucial for processing and applications, it is necessary to develop a feasible method to prepare water-soluble graphene-based flame retardants. In contrast to chemical modification, which is often based on covalent linkages between GO and flame-retardant compounds, non-covalent modification has many advantages such as high efficiency and easy preparation process [38–40]. More importantly, non-covalent bonding through *π*–*π* interactions never degrade the physical and chemical properties of GO [41]. In this way, non-covalently functionalized GO can be readily fabricated in aqueous solutions.

Since it was first proposed by Decher et al. [42], layer-by-layer (LBL) technique has proven to be very useful for assembling oppositely charged materials into thin films or coatings for membranes used in nanofiltration [43, 44], photo-catalysis [45], and controlled molecular release [46]. GO contains a substantial amount of oxygen groups and exhibits a negative charge when dispersed in water, forming a suspension that can be used to prepare a thin GO film by the LBL self-assembly technique. In particular, this unique 2D structure of GO offers an exciting opportunity to create LBL membranes by stacking GO nanosheets, which can be used to fabricate multifunctional hybrid films with nanometer precision [47–52]. Only a few studies deal with the preparation of LBL GO films or coatings for flame-retardant applications [53–55].

In this work, functionalized GO was prepared through non-covalent *π*–*π* stacking interactions with a flame-retardant compound containing phosphorus and nitrogen elements. The LBL-assembled GO-based flame-retardant multilayer films were deposited on the surfaces of poly(vinyl alcohol) (PVA) films with anionic functionalized GO and cationic polyethyleneimine (PEI). Thermal stability and flame-retardant properties of the coated PVA were studied systematically, and a detailed analysis of the mechanism is reported.

## Experimental

### Materials

Graphite powder was kindly supplied by Nanjing XFNano Materials Tech Co., Ltd. Potassium permanganate (KMnO_4_), sulfuric acid (H_2_SO_4_, 98%), hydrogen peroxide (H_2_O_2_), and sodium nitrate (NaNO_3_) were purchased from Aladdin Chemical Co., Ltd. Phenoxycycloposphazene (HPTCP) was friendly provided by Shengyi Technology Co., Ltd. PEI was purchased from Aladdin Chemical Co., Ltd. and PVA, with a polymerization degree of 1700 and an alcoholysis degree of 99, was supplied by Sichuan Vinylon Corporation (Chongqing, China). The deionized (DI) water used was prepared in our laboratory.

### Preparation of Non-covalently Functionalized GO

**Fig. 1:**
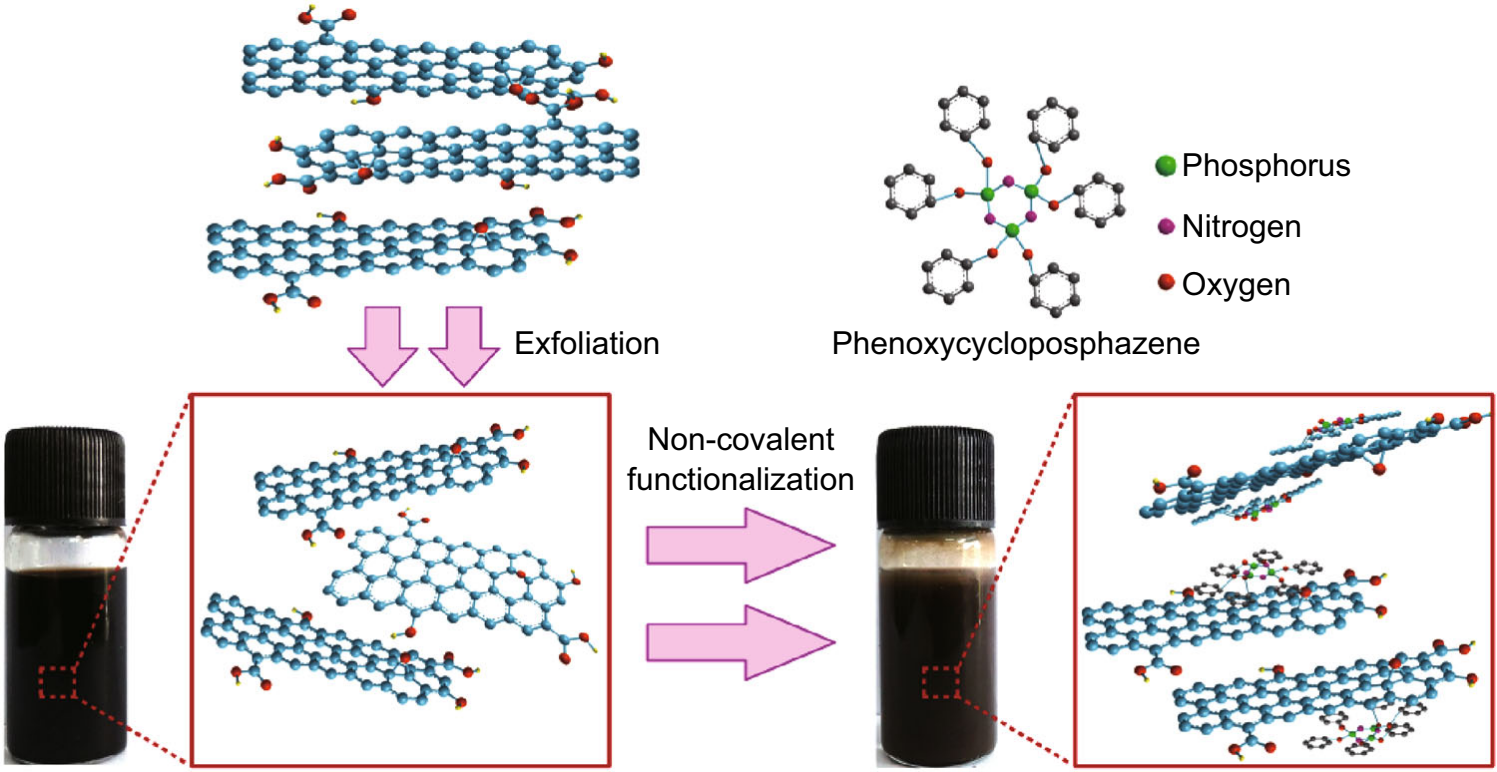
Schematic diagram showing overall processing of non-covalently FGO

GO was prepared from graphite using the modified Hummers method [56, 57]. The oxygen groups introduced into GO can effectively reduce the van der Waals forces between the neighboring carbon sheets, and water molecules can easily penetrate the interlayer of GO. Thus, GO can be exfoliated into single-layer sheets in water with the assistance of mild sonication. The photograph in Fig. 1 shows the GO (200 mg) forming a stable colloidal suspension in 200 mL of DI water after exfoliation. Subsequently, 20 mL of alcoholic solution of HPTCP (200 mg) was dropped into the GO suspension under stirring. HPTCP became less soluble in water due to the difference between the polarities of water and ethanol. Therefore, the less-soluble HPTCP was attached to GO via strong *π*–*π* interactions. Continuous penetration of HPTCP molecules into the GO sheets helped in achieving a more stable dispersion of FGO in water, and the *π*–*π* interactions prevented the exfoliated sheets from restacking.

### Preparation of PVA Film Coated with FGO-Based Multilayer

Designing oppositely charged suspensions is crucial for successful LBL assembly. Intrinsically, GO is negatively charged when dispersed in water because of the existence of oxygen-containing groups on its surface. The LBL deposition process was used to distribute PEI and FGO on the PVA membranes. The PVA films were immersed into a PEI aqueous solution (2 mg mL^−1^) for 5 min, followed by 1 min of rinsing in a purified water bath and subsequent drying at 80 °C for 10 min under vacuum to remove the solvent. The prepared PEI-modified PVA membranes were then immersed into the FGO aqueous solution (2 mg mL^−1^) for 5 min, followed by the same rinsing and drying steps as mentioned above. These steps were repeated until the desired number of bilayer (FGO/PEI) was deposited on the PVA substrate.

### Characterization

The morphology and structure of GO and FGO were studied by transmission electron microscopy (TEM) using a Tecnai G2 F20 electron microscope at an accelerating voltage of 200 kV. X-ray diffraction (XRD) patterns were recorded using a DX-1000 diffractometer (Dandong Fangyuan Instrument Co., Ltd., China) with a Cu-K_*α*_ generator system operated at 40 kV and 25 mA over a 2*θ* range of 5°–40° at a scanning rate of 1° s^−1^. Raman spectroscopy was conducted on a Labram HR spectrometer (HORIBA Jobin–Yvon) using 532-nm laser excitation with a power of 1 mW. Thermogravimetric analysis (TGA) was performed using a TA Q-500 TGA thermal analyzer at a heating rate of 10 °C min^−1^ over the temperature range of 30–650 °C with a nitrogen flow of 100 mL min^−1^. Approximately 8–10 mg of the sample was used in each test. UV–Vis absorption measurements were taken using a UV–visible spectrophotometer (Cary 100 Bio, Varian, USA). The surface morphology of the samples was observed using a scanning electron microscope (SEM) (JSM-5900LV, JEOL Ltd., Tokyo, Japan) with a conductive gold coating at an accelerating voltage of 10 kV. Microscale combustion calorimetry (MCC) analysis was carried out using an FAA-PCFC microscale combustion calorimeter (Fire Testing Technology Limited, UK) by heating about 2 mg of samples from ambient temperature to 800 °C at a heating rate of 1 °C s^−1^ under air atmosphere. Vertical burning tests of the samples were conducted on a HK-HVR vertical burning tester (Zhuhai Huake Testing Equipment Co., Ltd.). Elemental compositions of the coating residue were studied using a Shimadzu/Kratos AXIS Ultra DLD multifunctional X-ray photoelectron spectrometer (Manchester, UK).

## Results and Discussion

### Structural and Properties of Non-covalently Functionalized GO

**Fig. 2:**
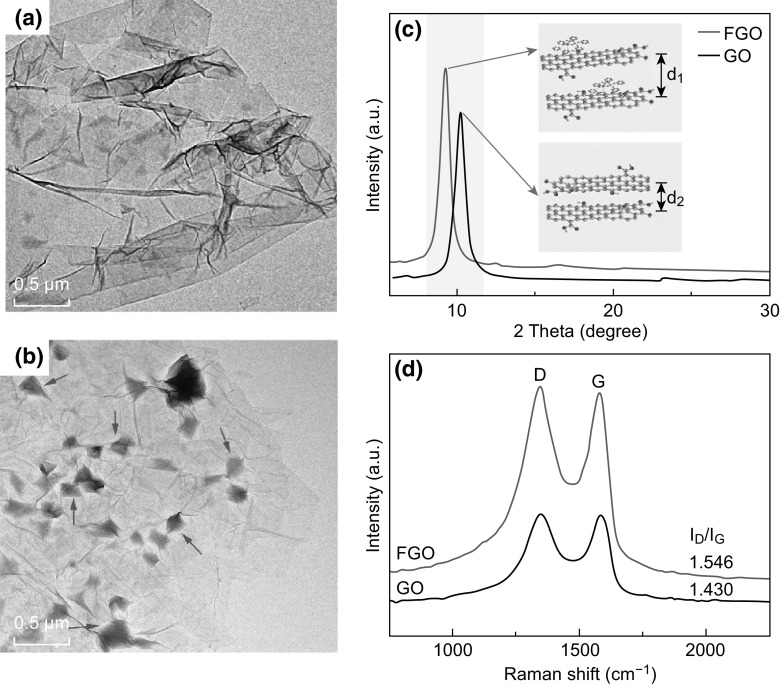
TEM images of **a** GO and **b** FGO. **c** XRD patterns and **d** Raman spectra of GO and FGO

The TEM images of GO and FGO are shown in Fig. 2a, b, respectively. A relatively large (about several micrometers) and uniform platelet with few wrinkles and protrusions is observed. It is revealed that the GO is composed of large single or few-layered sheet. In FGO, the layered structure of GO is not destroyed and the attached HPTCP molecules are clearly visible on the GO surface. In addition, many cracks appeared due to the non-covalent interactions between GO and HPTCP.

The XRD patterns of GO and FGO are shown in Fig. 2c. The typical diffraction peak at 2*θ* = 10.4° was assigned to GO, indicating an interlayer distance of 0.85 nm (*d*_2_), which is in good agreement with previous results [58]. After non-covalent functionalization, the peak shifted to 2*θ* = 9.2° in the FGO spectrum, suggesting that the interlayer distance increased to 0.96 nm (*d*_1_). As shown in Fig. 2c, the increase in interlayer distance (from *d*_2_ to *d*_1_) is due to the successful loading of HPTCP molecules on the surface of GO. Raman spectroscopy was conducted to further investigate the corrugated structures of GO and FGO. As shown in Fig. 2d, in-phase vibration of the sample’s lattice (G band) at 1570 cm^−1^ and the disorder band (D band) at approximately 1355 cm^−1^ was detected in the Raman spectra [59]. FGO exhibits a pattern similar to GO; thus, the non-covalent modification did not destroy the layered structure of GO. The intensity ratio of the D and G bands is also a key parameter for evaluating the structure of graphene. The *I*_D_/*I*_G_ ratios were 1.430 and 1.546 for GO and FGO, respectively. The slight increase in *I*_D_/*I*_G_ ratio of the latter indicates an increase in amorphous carbon compared to the *sp*^2^-hybridized graphene due to the loading of HPTCP.

**Fig. 3:**
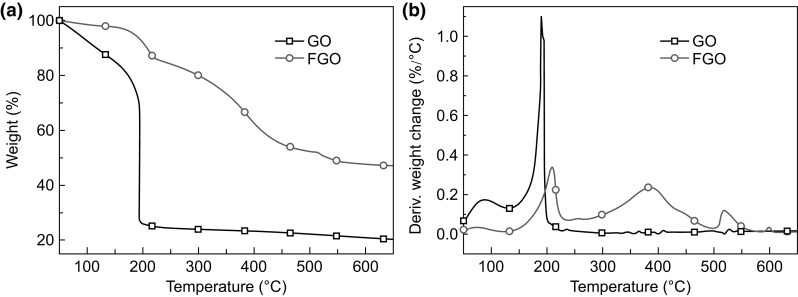
TGA profiles of GO and FGO under nitrogen atmosphere

TGA was employed to evaluate the thermal stability of GO and FGO. As shown in Fig. 3, GO showed a weight loss of approximately 70% below 200 °C due to the evaporation of residual moisture and decomposition of labile oxygen-containing functional groups. This indicates that GO cannot used alone as the effective flame retardant for polymers. The thermal stability of FGO was significantly higher than that of GO. The weight loss above 300 °C in the TGA profile of FGO predominantly belongs to the decomposition of HPTCP (Fig. S1). Importantly, the residual weight of FGO at 600 °C was 47.6%, which is more than two times that of GO (20.9%).

### Structural and Properties of PVA Film Coated with FGO-Based Multilayer

**Fig. 4:**
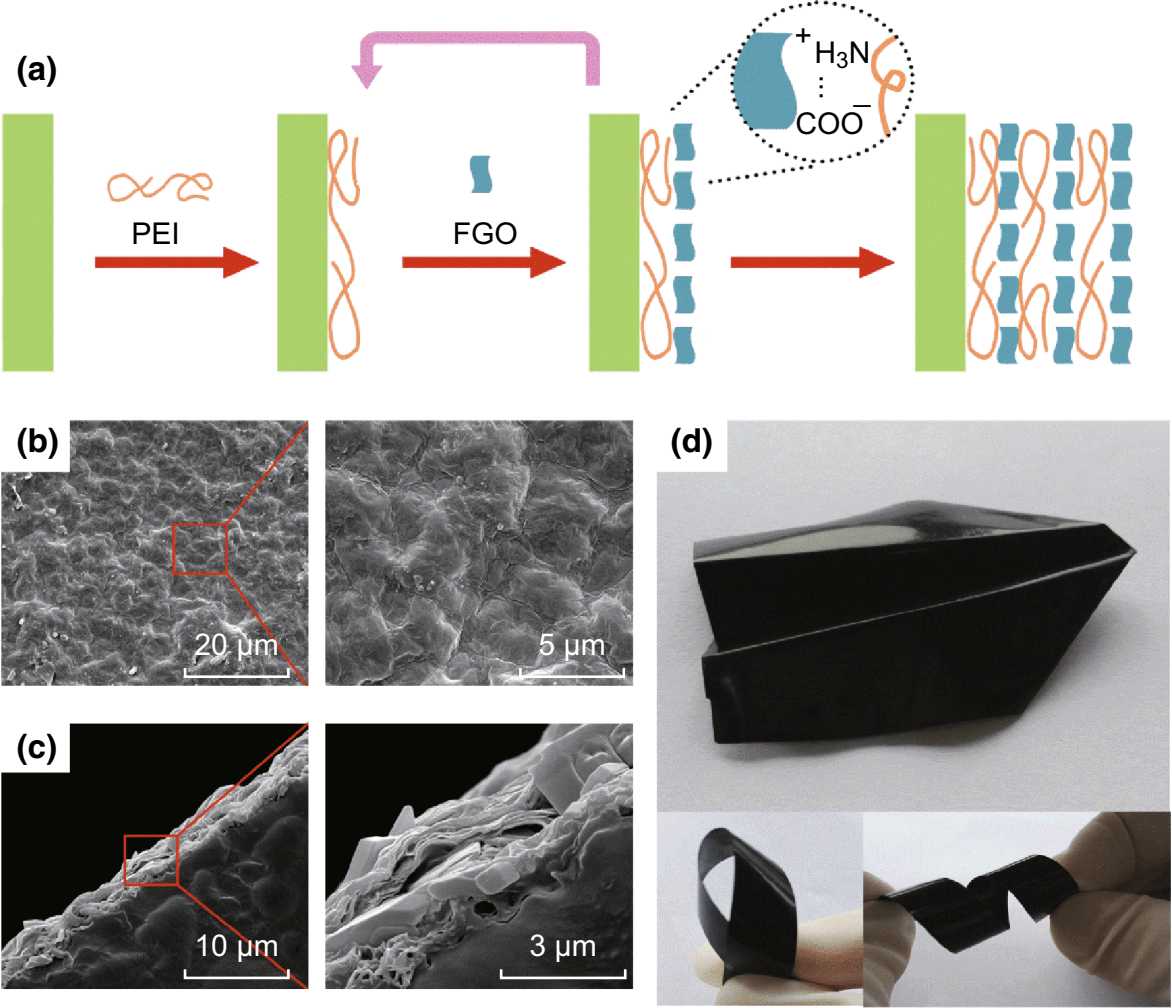
**a** Schematic diagram of LBL assembly of FGO/PEI bilayers on PVA film. **b** SEM top-view images and **c** cross-sectional images of the coated sample. **d** Photographs showing excellent flexibility of the coated PVA film

Figure 4a shows the schematic of the fabrication procedure of coated PVA film based on FGO/PEI bilayers via water-based LBL assembly. A cleaned PVA film was alternately dipped into oppositely charged PEI and FGO solutions, allowing electrostatic deposition of FGO sheets on the surface of the PVA film. The morphology and structure of the prepared LBL-assembled FGO/PEI multilayers were characterized by SEM, as shown in Fig. 4b, c. The PVA substrate was covered with sheet-shaped folds, which confirmed the successful assembly of the FGO layer. A layered structure is evident from the cross-sectional images due to the uniformly stacked FGO and PEI layers, and the thickness of the coating increased gradually with increasing number of self-assembled layers, as shown in Fig. S2. Besides, the relative mass of FGO/PEI coating to that of the PVA film increased with increasing LBL deposition cycles (shown in Fig. S3), indicating the effectiveness of coating via LBL assembly by using oppositely charged suspensions. After deposition of the LBL multilayer, the coated PVA film retained its original strength and toughness. Thus, the coated sample showed excellent flexibility during bending or twisting and could even be folded into a variety of shapes like a paper, as shown in Fig. 4d.

**Fig. 5:**
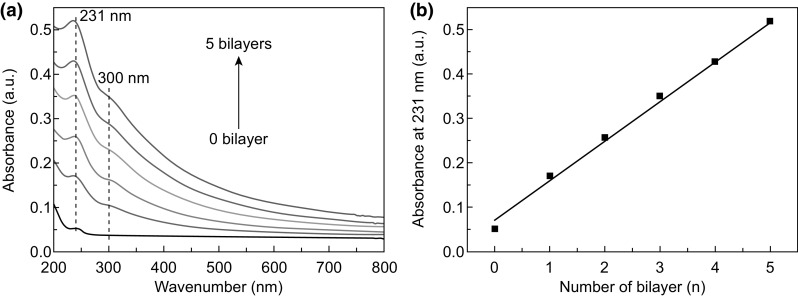
**a** UV–Vis spectra of GO-based multilayers deposited on PVA substrate and **b** absorption intensity at 231 nm as a function of bilayer number

UV–Vis absorption spectroscopy was used to monitor the coating growth on the PVA substrate. As shown in Fig. 5a, the main absorption in the UV–Vis spectral range of 200–350 nm increased with increase in the number of GO-based bilayers. The absorption peaks at 231 and 300 nm can be attributed to the *π*–π* transitions of aromatic C–C bonds and *n*–π* transitions of C=O bonds [60, 61], respectively. Besides, the linear increase in absorption intensity of the 231-nm peak confirmed that the deposition via LBL assembly is uniform, as can be seen in Fig. 5b.

**Fig. 6:**
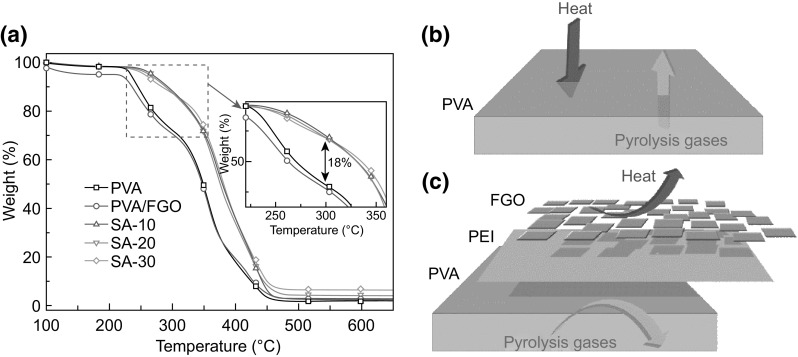
**a** TGA profiles of neat PVA, PVA/FGO, and PVA coated with different FGO-based layers. Schematic depicting degradation processes of **b** pure PVA and **c** coated PVA

The mass loss profiles of PVA, PVA/FGO composites and coated PVA are displayed in Fig. 6a. It should be noted that we refer to the self-assembled PVA/(FGO/PEI)_*n*_ sample as SA-n such as SA-10, SA-20, and SA-30. The main degradation of neat PVA took place between 250 and 300 °C, followed by a further weight loss between 350 and 450 °C. As reported in the previous literature [62, 63], these two degradation stages are attributed to the elimination of side groups at a lower temperature and the breathing of the polymer backbone at a higher temperature, respectively. PVA/FGO exhibited a similar weight-loss profile as that of neat PVA, except for a slight drop caused by the decomposition of FGO below 200 °C. When PVA was coated with the LBL-assembled FGO-based multilayer film, the thermal stability was significantly enhanced for all the coated PVA samples. From the corresponding data in Table 1, 5% weight loss of coated PVA occurred at about 260 °C, which is over 30 °C higher than the weight-loss temperature of neat PVA. Huang et al. [64] reported the initial decomposition temperature of flame-retardant PVA/graphene composite as 17 °C higher than that of pure PVA. In addition, compared to PVA/FGO composite with the same loading content of FGO, the coated PVA (SA-30) showed a higher residual content at 600 °C, which is more than two times that of the former. It can be concluded that delay in the thermal degradation of PVA and enhancement in the char formation can be ascribed to the excellent physical barrier effect provided by the FGO-based multilayer. The proposed mechanism of enhanced thermal stability is depicted in Fig. 6c. During the heating process, the compact FGO/PEI multilayer acted as a protective coating, blocking the transfer of heat and pyrolysis gas and thus shifting the degradation temperature of PVA to higher temperatures.

**Table 1:** TGA data of neat PVA, PVA/FGO and coated PVA samples

Sample	Temperature at 5% weight loss (°C)	Residual content at 600 °C (%)
PVA	234	1.51
PVA/FGO	216	2.97
SA-10	266	2.16
SA-20	262	4.18
SA-30	257	6.39

### Flame Retardancy and Mechanism of PVA Film Coated with FGO-Based Multilayer

**Fig. 7:**
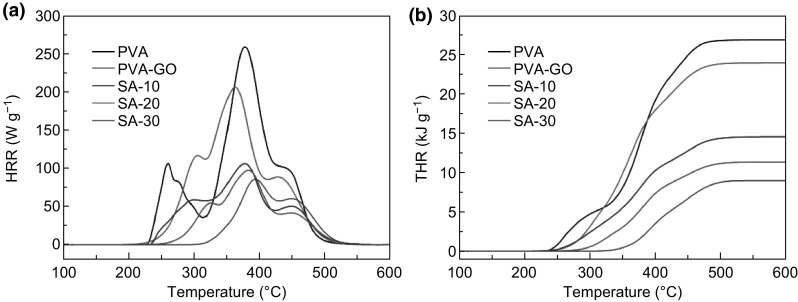
**a** HRR and **b** THR profiles of pure PVA, PVA/GO, and self-assembled FGO-coated PVA with different numbers of bilayers

MCC test was performed to evaluate the fire-retardant property of the prepared samples. Figure 7 shows the typical heat release rate (HRR) and total heat release (THR) profiles of neat PVA, PVA/GO, and FGO/PEI-coated PVA films. As reported in the literature [23], GO sheets are easily combustible; thus, individual GO incorporated into a PVA film has almost no effect on the heat release behavior of the PVA/GO composite. Compared to pure PVA, the FGO/PEI-coated PVA achieved significant improvements in flame resistance, including a decrease in heat release and an increase in the temperature of initial heat release. The peaks of heat release rate (PHRR) of all the coated PVA samples are lower than that of pure PVA and gradually decreased with increasing number of FGO-based bilayers. The temperature of initial heat release increased from 226 for pure PVA to 318 °C for PVA coated with 30 FGO bilayers. Besides, the THR of the coated PVA substantially reduced, and the THR value of SA-30 was 8.9 kJ g^−1^, which is 67% lower than that of pure PVA. As reported in Huang’s work [64, 65], the THR reduced by 69% and 19% for the PVA/graphene/melamine polyphosphate system and the PVA/graphene/montmorillonite system, respectively. Accordingly, the obtained excellent fire retardancy is probably attributed to the creation of a barrier effect on the surface of PVA, which can decelerate the heat transfer and prevent the underlying materials from further combustion. Further, the effect of PEI on flame retardancy was evaluated using the MCC test. The FGO-coated PVA film was prepared by adsorbing FGO sheets on the PVA surface through strong *π*–*π* stacking interactions. It can be seen from Fig. S4 that the FGO-coated PVA exhibits a slightly lower heat release than the FGO/PEI-coated PVA does with an equal amount of deposition. However, the coated PVA film becomes extremely brittle after several deposition cycles of GO without successively incorporated PEI layers.

**Fig. 8:**
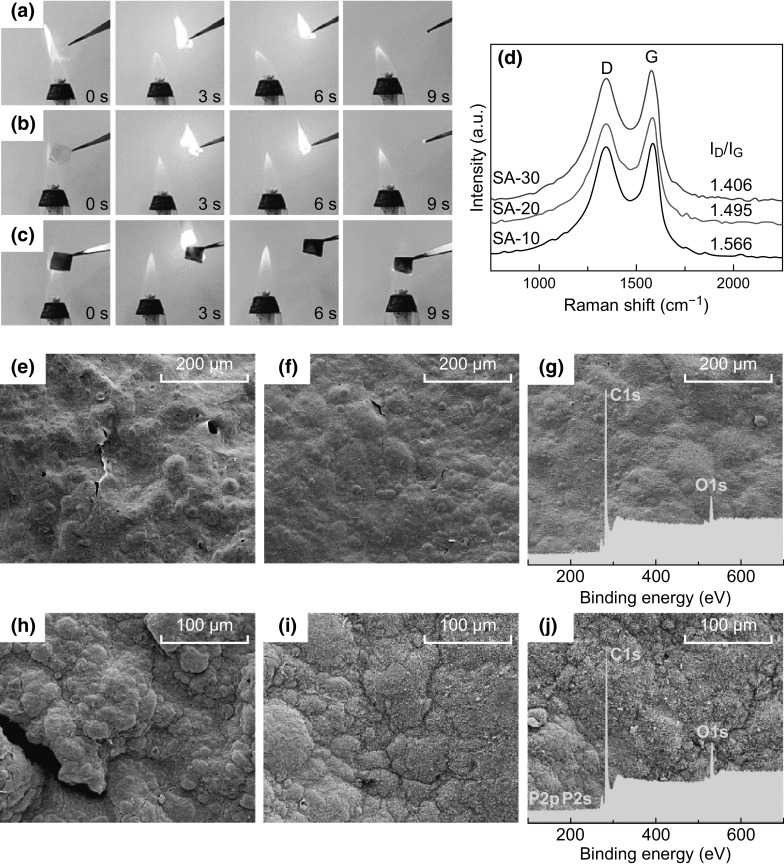
Snapshots of flame treatment of **a** PVA film, **b** PVA/FGO composite film, and **c** LBL-assembled FGO-coated PVA film with 10 bilayers with respect to time in 9 s. **d** Raman spectra of PVA coated with different self-assembled (FGO/PEI) bilayers after flame treatment. SEM images of external surfaces of FGO-based coating residue with **e** 10, **f** 20, and **g** 30 bilayers and internal surfaces of FGO-based coating residue with **h** 10, **i** 20, and **j** 30 bilayers. The insert shows XPS spectra of external and internal surfaces of FGO-based coating residue

We investigated the flame-retardant properties of pure PVA, PVA/FGO composite film, and the LBL-assembled FGO-coated PVA film by direct exposure to an ethanol flame. The images captured from the combustion video with an interval of 3 s are shown in Fig. 8a–c. The pure PVA sheet initially burned instantly and left almost no residue after combustion. The PVA/FGO composite sheet also burned out quickly, indicating that the evenly dispersed FGO does not exert its barrier effect in the condensed phase. Interestingly, the LBL-assembled FGO-coated PVA sheet remained inert and preserved its shape with little shrinkage even after repeated exposed to high-temperature flame for 9 s, as shown in Fig. 8c. The vertical burning test was conducted to evaluate the combustion behaviors of the different samples, as shown in Fig. S5. The excellent flame-retardant properties arose mainly due to the formation of a high-quality barrier layer, which is based on the graphitic char from GO and the HPTCP-catalyzed char from the PVA matrix. Raman spectroscopy is commonly used to characterize the structures of carbonaceous materials. Figure 8d shows the Raman spectra of the char residues of the coated PVA samples. The Raman spectra of all the residual char exhibited the G band at 1570 cm^−1^ and the D band at 1355 cm^−1^. A lower *I*_D_/*I*_G_ ratio implies higher graphitization degree and thermal stability of the char structure [22, 66]. The *I*_D_/*I*_G_ ratios of the char residues gradually decreased with an increase in the number of FGO-based bilayers. In other words, a large amount of graphitized carbon was formed in the residual char, which provided a protective shield to block the heat and mass transfer between the flame and the material.

The self-assembled FGO-based multilayer converted into an integrated char layer coating the PVA substrate after the flame treatment. We carefully cut the char layer from the burned sample. The surface exposed to the flame is regarded as the external surface of the FGO-based coating residue, while the surface in contact with the PVA substrate is the internal surface. The SEM images of the coating residue shown in Fig. 8e–j reveal that a more dense and continuous char layer with fewer cracks and holes is obtained with increasing number of bilayers from 10 to 30. It is noteworthy that there is a significant difference between the morphologies of the external and internal surfaces of the coating residue. The external surface appeared relatively smooth with a sheet-shaped structure, while the internal surface exhibited continuous sheets covered with a large number of small particles. From the combined results of XPS analysis, it is revealed that the outer layer is mainly composed of C and O elements, with additional P element observed in the inner layer of the coating residue. Accordingly, it is concluded that two different char structures were generated by different processes: the outer layer is a physical char mainly consisting of graphene sheets, while HPTCP catalyzed the PVA matrix into a chemical char that encapsulated the physical char, thus constituting the inner layer.

**Fig. 9:**
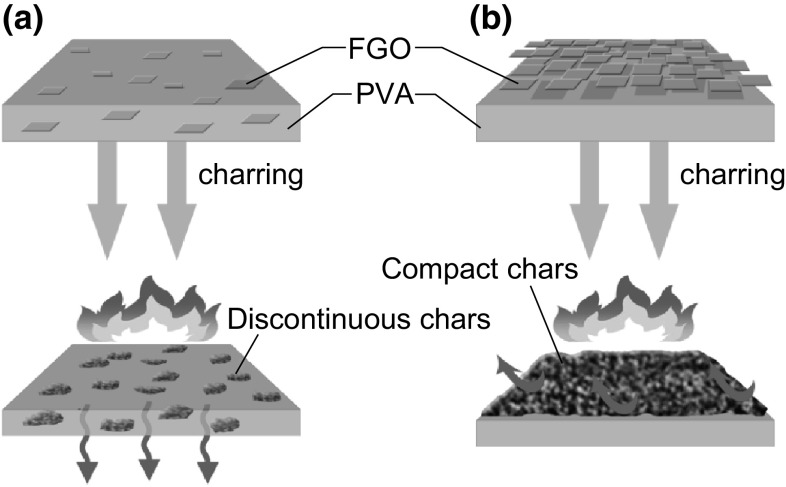
Scheme of the flame-retardant behaviors of **a** PVA/FGO composite and **b** FGO multilayer-coated PVA

Owing to the different flame-retardant behaviors as illustrated in Fig. 9, the char generated from the self-assembled FGO coating enriched the surface of the PVA matrix, rather than being non-uniformly distributed over the entire matrix. Apparently, the coating char exerts a much greater isolating effect on flammable volatiles, oxygen, and heat than the independent char formed in bulk does, thus showing significantly improved flame retardancy compared to the latter with the same flame-retardant content.

**Fig. 10:**
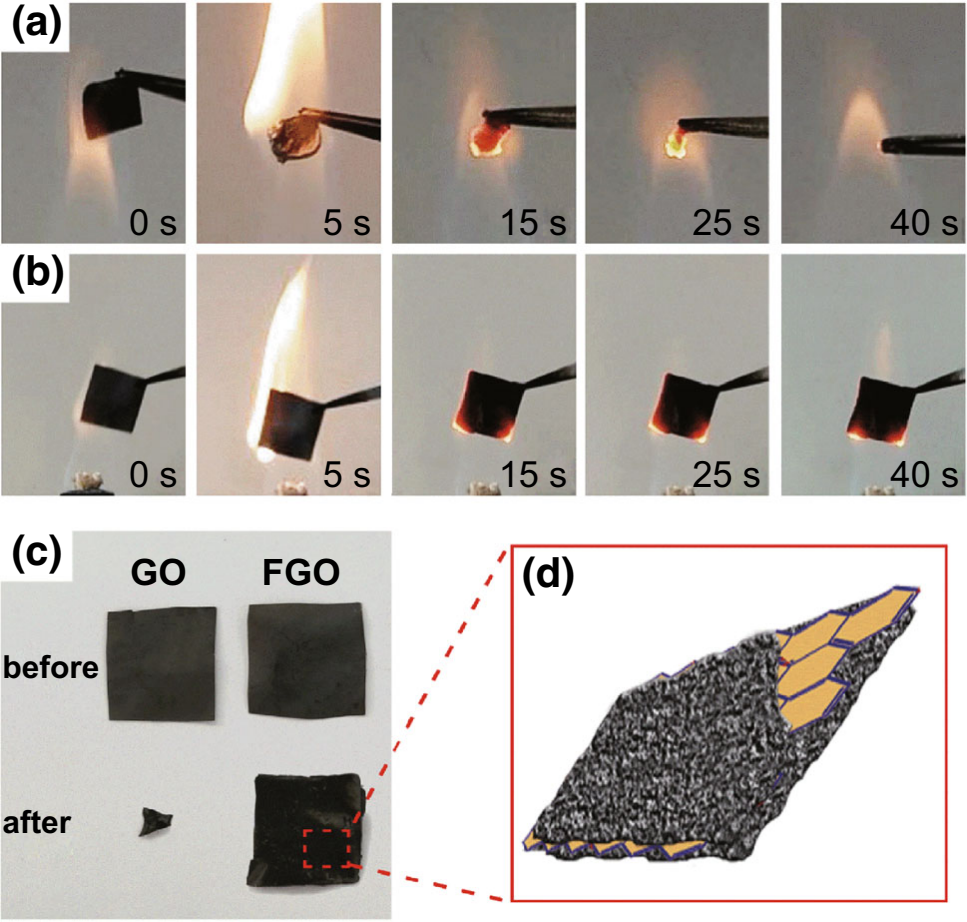
Snapshots of flame treatment of PVA sheet coated with 30 LBL-assembled bilayers based on **a** GO and **b** FGO with respect to time in 40 s. **c** Photographs showing morphology of films before and after flame treatment. **d** Schematic diagram of char structure of FGO-based residue layer

As is well known, GO exhibits an extremely low thermal stability and easily combusts in air atmosphere owing to abundant oxygen-containing groups, severely restricting its application as flame retardants. Here, we comparatively studied the flame-retardant properties of coated PVA sheet and those of GO-based and FGO-based multilayers. Figure 10 shows the snapshots of the flame treatment process. Upon exposure to flame, the GO-based multilayer-coated PVA sheet started to curl and shrink, and the entire part turned red hot in 15 s. The shrinkage tended to increase with prolonged treatment time, and the sheet completely burned off in 40 s. On the other hand, the FGO-based multilayer-coated PVA sheet maintained its initial shape without any shrinkage and without catching fire within 15 s. Even after 40 s, there was no further change in the shape of the FGO-coated PVA sample, as shown in Fig. 10c. Accordingly, the char structure of the FGO-based residue illustrated in Fig. 10d can be identified as a composite char, comprising the physical char from graphene and the chemical char from the catalyzed PVA matrix. These results indicate that the incorporation of HPTCP on the GO sheets plays a critical role in the construction of a compact flame-retardant layer to block heat and oxygen transfer, demonstrating that FGO-based LBL coatings can serve as an effective flame-retardant treatment for polymer materials.

## Conclusion

In summary, we fabricated a novel type of flame-retardant membrane by successively assembling negatively charged non-covalent FGO nanosheets and positively charged PEI on a PVA support based on the electrostatic LBL self-assembly technique. FGO was prepared by incorporating HPTCP onto the surfaces of GO sheets via a non-covalent strategy, thus facilitating the barrier effect of GO in the condense phase. HPTCP molecules were successfully loaded onto the GO surface without affecting its original structure and property. After a uniform deposition process, a compact and continuous multilayer coating based on FGO/PEI was constructed on the PVA surface. The initial decomposition temperature of the coated PVA improved by more than 30 °C compared to that of pure PVA, showing significantly enhanced thermal stability due to the effective barrier effect of the stacked FGO sheets. In addition, we evaluated the flame retardancy of the coated samples and a corresponding mechanism was proposed. The results indicate a substantial reduction in heat release for coated PVA, and the 30-bilayer-coated sample maintained its initial shape even after prolonged direct exposure to flame. A composite char, consisting of the physical char from graphene and the chemical char from the catalyzed matrix, formed on the PVA surfaces as a protective shield to effectively block heat and mass transfer. Therefore, the reported FGO-based LBL coating may have great potential applications in the flame-retardant treatment of various polymers.

## Electronic supplementary material

Below is the link to the electronic supplementary material.

Supplementary material 1 (PDF 540 kb)
