# Supplementary material for: Non-covalently Functionalized Graphene Oxide-Based Coating to Enhance Thermal Stability and Flame Retardancy of PVA Film
Source: Nanomicro Lett. 2018 Feb 16;10(3):39. doi: 10.1007/s40820-018-0190-8 (PMC6199081; doi:10.1007/s40820-018-0190-8)
Supplement: Supplementary file 1 — Supplementary material 1 (PDF 540 kb) [file 40820_2018_190_MOESM1_ESM.pdf]

Supporting Information for

Non-covalently Functionalized Graphene Oxide based Coating to Enhance Thermal Stability and Flame Retardancy of PVA Film

Wenhua Chen[1,#], Pengju Liu[1,#], Lizhen Min[1], Yiming Zhou[1], Yuan Liu[1,1], Qi Wang[1], Wenfeng Duan[2]

[1]State Key Laboratory of Polymer Materials Engineering, Polymer Research Institute of Sichuan University, Chengdu 610065, People’s Republic of China

[2]State Key Laboratory of Special Functional Waterproof Materials, Beijing Oriental Yuhong Waterproof Technology Co. Ltd., Beijing 101300, People’s Republic of China

[#]These authors contributed equally to this work

[1]Corresponding author. E-mail: liuyuan42001@163.com (Yuan Liu)

Supplementary Results and Discussions

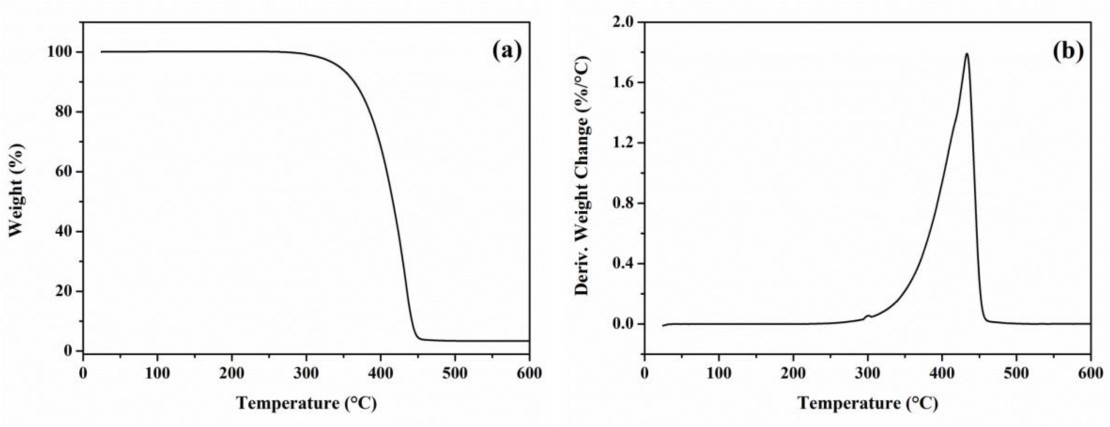

Fig. S1 a mass loss curve and **b** corresponding derivative curve of Phenoxy-cycloposphazene under nitrogen atmosphere

TGA was performed to study the decomposition process of HPTCP and the corresponding curves were shown in Fig. S1. The initial decomposition temperature (temperature at 5 wt% weight loss) and the main decomposition temperature of HPTCP were about 345 and 430 °C, respectively.

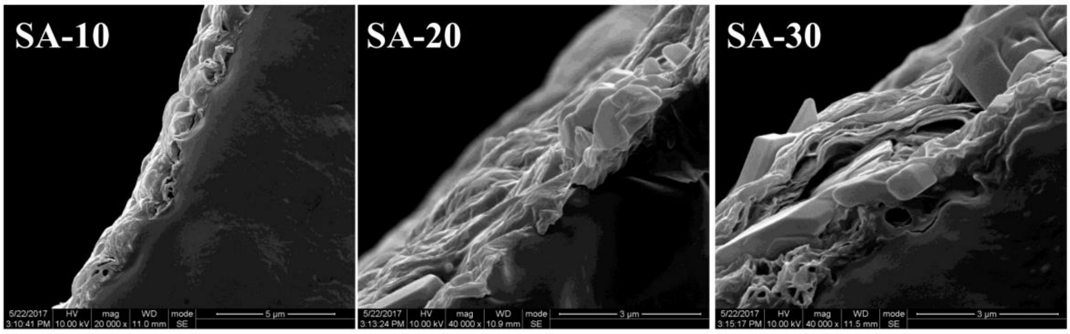

Fig. S2 Cross section images of the coated sample with different bilayers

Resulting from the uniformly stacked FGO and PEI layers, a clear layered structure was observed from the cross-section images. The boundaries between neighbor layers may be relatively indistinct, but the length of the coating increases gradually with increasing the self-assembled layers as shown in Fig. S2.

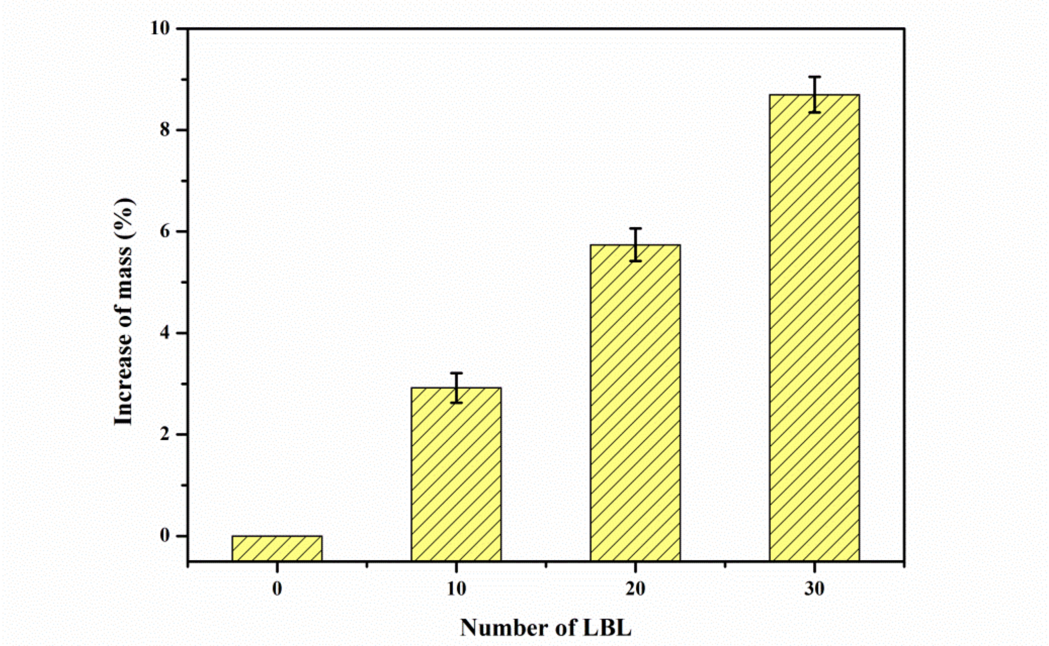

Fig. S3 Relative increase of coating mass on PVA film

It can be seen from Fig. S3, the relative mass of FGO/PEI coating to PVA film also increased with increasing LBL deposition cycles, indicating the effectiveness of coating via LBL assembly by using oppositely charged suspensions.

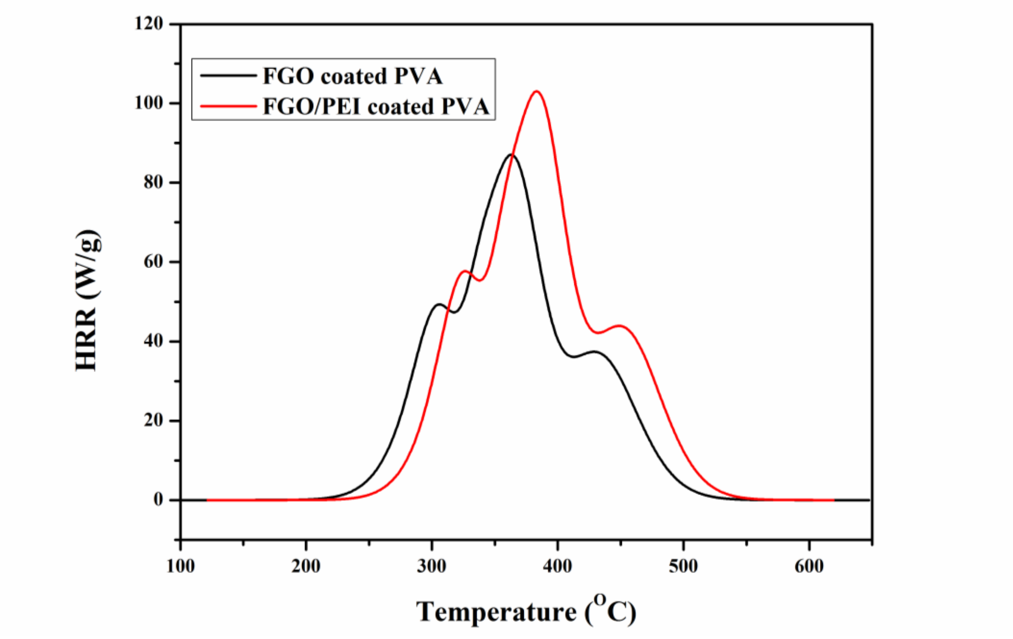

Fig. S4 Heat release curve of FGO coated PVA and FGO/PEI coated PVA samples with the same amount of loading

The FGO can deposit on the PVA surface through a strong pi-pi stacking interaction. And then the flame retardancy of FGO coated PVA and FGO/PEI coated PVA samples were comparatively studied using MCC test. With the same increase of mass, the FGO coated PVA sample exhibits a slightly lower heat release than FGO/PEI coated one. However, the coated PVA film will become extremely brittle after 10 deposition cycles of GO without successively incorporating PEI layers. Thus, PEI is necessary to fabricate the coated PVA film with good comprehensive performance.

We adopted the standard vertical burning test (UL94) to comparatively evaluate the flame retardancy of different samples, and the related data was shown in Fig. S5. The pure PVA and PVA/FGO composite film had a very fast combustion rate without flame extinguishing. However, for FGO coated PVA film, it could self-extinguish after the first ignition although a certain degree of deformation occurred. And then the coated PVA film would never catch the fire when further exposed in the flame, showing the excellent flame resistance property.

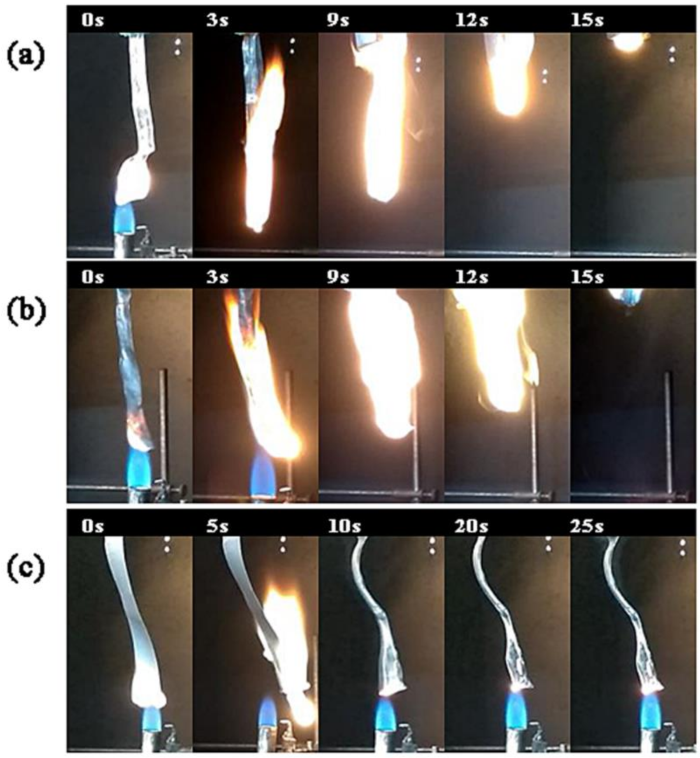

Fig. S5 Vertical burning test of **a** PVA film, **b** PVA/FGO composite film and **c** LBL assembled FGO coated PVA film with 30 bilayers
